# Soil temperature and hydric potential influences the monthly variations of soil *Tuber aestivum* DNA in a highly productive orchard

**DOI:** 10.1038/s41598-019-49602-2

**Published:** 2019-09-10

**Authors:** Flora Todesco, Simone Belmondo, Yoann Guignet, Liam Laurent, Sandrine Fizzala, François Le Tacon, Claude Murat

**Affiliations:** 1 Université de Lorraine, INRA, UMR IAM, Lab of Excellence ARBRE, 54000 Nancy, France; 2 Chambre d’Agriculture de la Charente, ZE ma Campagne, 16016 Angoulême Cedex, France

**Keywords:** PCR-based techniques, Fungal ecology

## Abstract

*Tuber aestivum*, also known as the summer or Burgundy truffle, is an ectomycorrhizal Ascomycete associated with numerous trees and shrubs. Its life cycle occurs in the soil, and thus soil parameters such as temperature and water availability could influence it. *T*. *aestivum* cultivation has started in several countries, but ecological and agronomic requirements for the establishment and management of orchards are largely unknown. The aims of this work were: 1) to design a specific qPCR protocol using genomic data to trace and quantify *T*. *aestivum* DNA in the soil; and 2) to assess the monthly soil DNA dynamic according to soil parameters (i.e. soil hydric potential and temperature) in this orchard. The study was conducted in a highly productive *T*. *aestivum* orchard (hazels, oaks, pines, lime and hornbeam). The production started five years after the plantation and then increased exponentially to reach a maximum of 320 kg/ha in 2017. The soil hydric potential and temperature partially explained the monthly *T*. *aestivum* soil DNA variability. The data presented here offer new insights into *T*. *aestivum* ecology and cultivation.

## Introduction

Ectomycorrhizal fungi, i.e., which live in symbiosis with tree and shrubs, play important roles in forest functioning and biogeochemical cycles^1^. In boreal forests, 50–70% of the carbon stored in the soil is derived from roots and root-associated microorganisms such as ectomycorrhizal fungi^2^. Besides forest ecosystems, ectomycorrhizal trees were also implanted in agroforestry ecosystems and in dedicated orchards for producing non-wood products such as edible fungi. The inoculation of tree seedlings with selected ectomycorrhizal fungi in nurseries (i.e., controlled mycorrhization) started hundred years ago, and this technique has been used extensively to grow truffles since the 1970s^3^.

True truffles (*Tuber* spp.) are ectomycorrhizal Ascomycetes producing hypogeous fruiting bodies. The genus *Tuber* is present globally in temperate areas including over 200 species^4,5^. Of these, at least 30 species are naturally present in Europe^6^, but only a few have remarkable organoleptic properties and social value^7–9^. The most valuable truffle species are *Tuber melanosporum* Vittad, the Périgord black truffle; *Tuber magnatum* Pico, the white Italian truffle; and *Tuber aestivum* Vittad, the summer and Burgundy truffle. Since the first commercialization of seedlings inoculated with *T*. *aestivum* and *T*. *melanosporum* in 1973, considerable progress has been made to improve the quality of the inoculated plants^3,10^. *T*. *melanosporum* plantations are found not only in the Mediterranean area but also outside their native range including Australia, New Zealand, South-Africa, South-America, and West USA^10^.

*T*. *aestivum* is the second most cultivated truffle species worldwide^3^. Versus *T*. *melanosporum*, it has the most extensive natural habitat from Morocco to Sweden and from Ireland to Azerbaijan^11–13^. Its wide natural distribution and long harvest season—make this species very promising for cultivation^12,14–16^.

The entire truffle life cycle occurs in the soil, and therefore soil parameters such as temperature and water availability could influence plantation success. However, information about ecological and agronomic requirements for the establishment and management of *T*. *aestivum* orchards (e.g. soil tillage, irrigation, and tree pruning) are largely unknown. To the best of our knowledge, there is no long-term data on *T*. *aestivum* ascocarps production in a plantation available in the literature. Molinier *et al*.^17^ published a study on the production of *T*. *aestivum* followed during 30-years, but this orchard used seedlings inoculated with *T*. *melanosporum*. Clear guidelines for orchard management are thus needed to improve cultivation of this species.

The genome of *T*. *aestivum* has been recently sequenced^18^. *T*. *aestivum* is a heterothallic species but it is impossible to realize its life cycle *in vitro*. To better understand its sexual reproduction cycle, an *in situ* small-scale genetic structure analysis was realized^19,20^. An important genotype turnover was observed suggesting a ruderal strategy. However, some genotypes spread several hundred meters. Gryndler *et al*.^21^ developed a qPCR protocol to better understand *T*. *aestivum* soil DNA localisation and dynamics. *T*. *aestivum* DNA was found profusely on the surface of roots other than ectomycorrhizae^21^. Moreover, stimulation of soil *T*. *aestivum* DNA with high doses of lime powder and other organic compounds (i.e. gallic acid, cellulose and calcium formate) was observed^22^. *T*. *aestivum* can share the same habitat as other truffle species (e.g. *T*. *melanosporum* and *T*. *mesentericum*) and thus it is possible that the DNA of all of these truffle species coexist in the soil^17,23^. This is an important issue because the Gryndler *et al*.^21^ protocol also amplified *T*. *mesentericum* DNA. Thus, it was necessary to develop another protocol that exclusively amplifies *T*. *aestivum* DNA.

Here, we analysed a highly productive *T*. *aestivum* orchard composed of 117 trees (hazels, oaks, pines, lime, and hornbeam) belonging to the French truffle grower’s federation experimental network. The aims of this study were: 1) to design a specific qPCR protocol via genomic data to trace and quantify *T*. *aestivum* DNA in the soil; and 2) to assess the monthly soil DNA dynamic over 24 months according to soil parameters (i.e. soil hydric potential and temperature).

## Results

### Evolution of the production in the experimental truffle orchard

**Figure 1:**
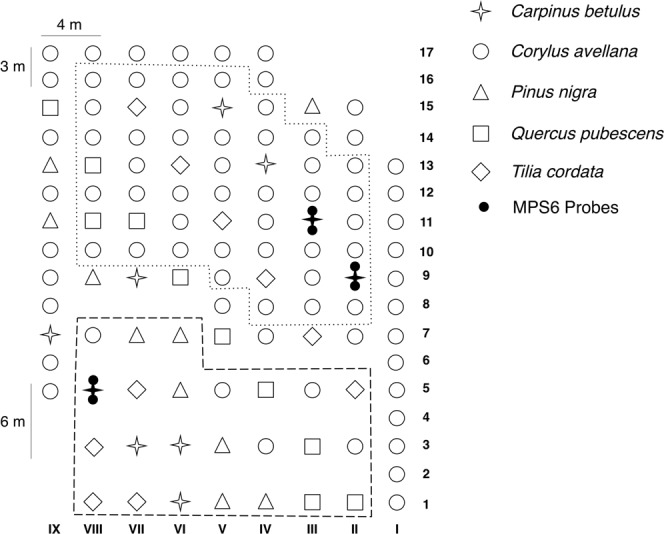
Map of the *T*. *aestivum* experimental orchards in Nouvelle-Aquitaine (France). Different symbols display the five different species within the site (117 trees in total). Stars, *Carpinus betulus* (10 plants); circles, *Corylus avellana* (77 plants); triangles, *Pinus nigra* (10 plants); squares, *Quercus pubescens* (10 plants); rhombus, *Tilia cordata* (10 plants). Seedlings of the five different species were planted randomly. Black stars represent the three highly producing trees (*C*. *betulus*) selected in the frame of the national experimental project Culturtruf (trees II-9, III-11 and VIII-5). Areas delimited by points and lines represent the high density (4 × 3 m equivalent to 833 trees per hectares) and low density (4 × 6 m equivalent to 417 trees per hectares) in the orchards, respectively.

**Figure 2:**
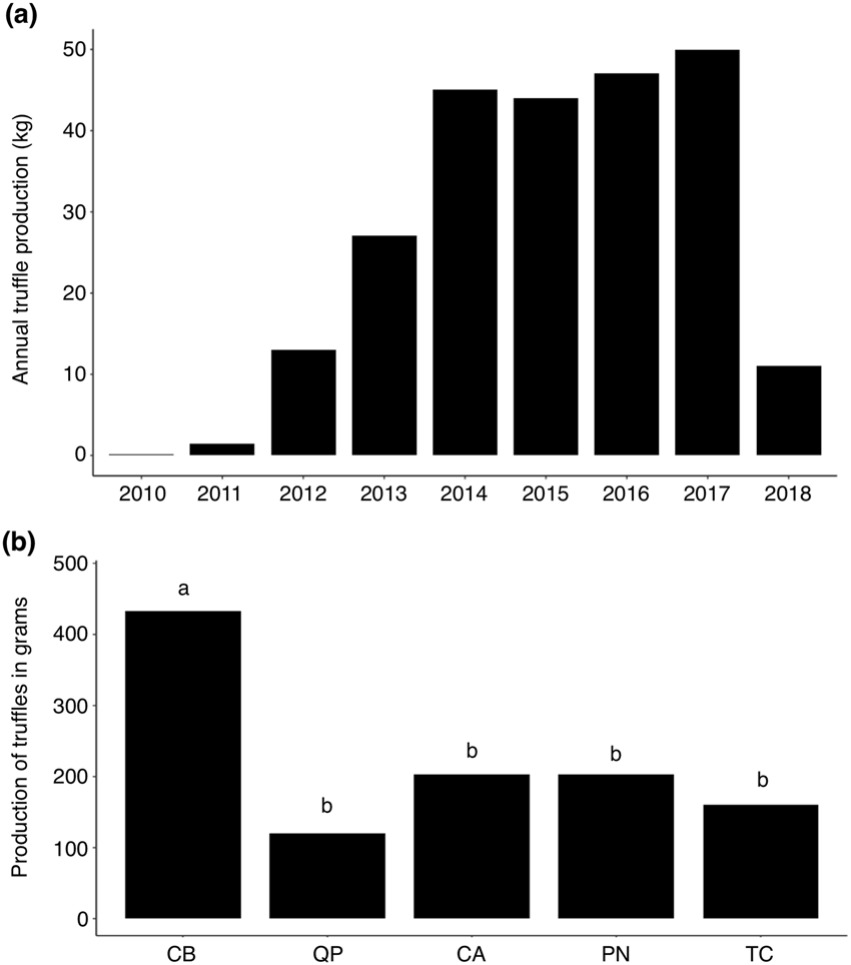
Evolution of the production in the experimental *T*. *aestivum* orchard. Annual total production in kg from 2009 to 2018 (**a**) and average production according to tree species from 2009 to 2018 (**b**). CB, *Carpinus betulus;* QP, *Quercus pubescens;* CA, *Corylus avellana; *PN, *Pinus nigra;* and TC, *Tilia cordata*. Different letters (**a**,**b**) indicate significant differences between tree species (Tukey’s HSD test, p < 0.05).

The production of the truffle orchard (Fig. 1) started in 2009—five years after the plantation (Fig. 2a and Supplementary Table S1). From 2009 to 2014 the annual *T*. *aestivum* production increased exponentially. The production then stabilized (about 40 kg corresponding to 266 kg/ha) to reach a maximum of 49 kg in 2017 (320 kg/ha). In 2018, the ascocarp production declined to 11 kg (73 kg/ha; Fig. 2a and Supplementary Table S1). We then compared the production of the five tree species present in the plantation. Considering the production from 2009 to 2018, the mean quantity of truffle harvested under *Corylus avellana*, *Pinus nigra*, *Quercus pubescens*, and *Ttilia cordata* was similar (between 100 to 200 g per tree), although it was significantly higher for *Carpinus betulus* (above 400 g per tree) (Figs 2b and S1).

### Evolution of the rainfall, soil hydric potential, and soil temperature

The daily rainfall, pF (i.e. soil hydric potential), and soil temperature recorded from September 2016 to September 2018 at north and south probes are presented in Supplementary Figs S2–S4 for *C*. *betulus* trees II-9, III-11, and VIII-5, respectively. The pF corresponded to the field capacity for all trees from November 2016 to March 2017 and from November 2017 to March 2018. In 2017, the pF started to increase from April to November to reach pF 4 (i.e. temporary wilting point) several days for trees II-9 and III-11. There were few days above pF 4 due to rainfall or watering (Supplementary Figs S2 and S3). In 2018, the pF was higher in May than in 2017; it reached pF 4 over several days for trees II-9 and III-11 during July and August. In both 2017 and 2018, the pF did not reach the temporary wilting point for tree VIII-5 (Supplementary Fig. S4). The daily pF was significantly higher for the probe in the north than the probe in the south of trees II-9 and VIII-5 (Wilcoxon p-value < 0.001). In contrast, it was higher for the probe in the south than the probe in the north for tree III-11 (Wilcoxon p-value < 0.001).

**Figure 3:**
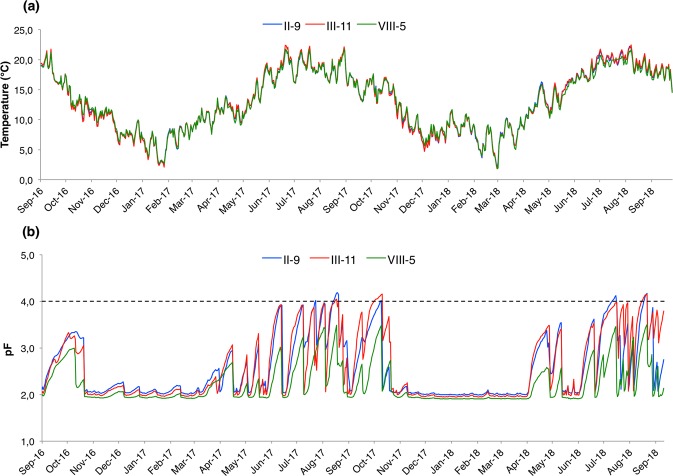
Graphic representations of temperature (**a**) and pF (**b**) (i.e. soil hydric potential) registered from September 2016 to September 2018 under tree II-9 (in blue), tree III-11 (in red), and tree VIII-5 (in green). The daily temperature and pF are represented as the average between north and south probes. The temporary wilting point (pF 4.2; black dashed line) is reached by tree II-9 and tree III-11 from June 2017 to October 2017 and from July 2018 to August 2018; this does not occur in tree VIII-5.

The maximum and minimum soil temperature was recorded in August 2018 with 22.7 °C for south probe of tree III-11 and in February 2018 with 1.7 °C for south probe of tree VIII-5, respectively (Supplementary Figs S3 and S4; Supplementary Tables S4 and S5). The daily temperature was significantly higher for the probe in the south than the probe in the north of trees II-9 and VIII-5 (Wilcoxon p-value < 0.01). In contrast, it was higher for the probe in the north than the probe in the south for tree III-11 (Wilcoxon p-value < 0.01). The daily pF and temperature recorded under tree VIII-5 were significantly lower than under trees II-9 and III-11 (Fig. 3; Wilcoxon p-value < 0.001 and <0.01, respectively).

### Development of a specific protocol to quantify DNA of *T. aestivum*

A total of 47,887 clusters were identified; 647 had one single copy gene model present only in *T*. *aestivum* (orphan genes). Clusters 17,691 and 17,699 correspond to gene models GSTUAT00001921001 (proteinId 4119) and GSTUAT00007989001 (proteinId 4108), respectively; these clusters were randomly selected. The gene models were called TuGM4119 and TuGM4108, respectively. TuGM4119 was 1542 nucleotides long, and coded for a protein with 428 amino acids and five exons. The NCBI database showed homology in 5′ with a calpain-like protein from *Trichoderma parareesei* (70 amino acids, e-value 2.10^−04^) and cystein protein of *T*. *magnatum* (152 amino acid, e-value of 4.10^−4^). There was no known Pfam motif.

TuGM4108 contained 626 nucleotides, and coded for a protein with 102 amino acid and two exons. It had no homology in the NCBI database or any Pfam motif. A pair of primers was designed in both gene models. PCR on thirteen DNA samples from different *Tuber* spp. samples showed no amplifications for primers TuGM4119f/TuGM4119r and amplification only for *T*. *aestivum* DNA for TuGM4108f/TuGM4108r (Supplementary Fig. S5). The primer pair TuGM4119f/TuGM4119r was not investigated further. In qPCR, TuGM4108f/TuGM4108r primers were amplified for all *T*. *aestivum* DNA tested but not in the DNA of other species (Supplementary Fig. S6a). Good amplification was seen in the qPCR of 16*T*. *aestivum* isolates harvested from different geographic location (Supplementary Fig. S6b).

### Temporal dynamic of *T. aestivum* DNA in the soil

**Figure 4:**
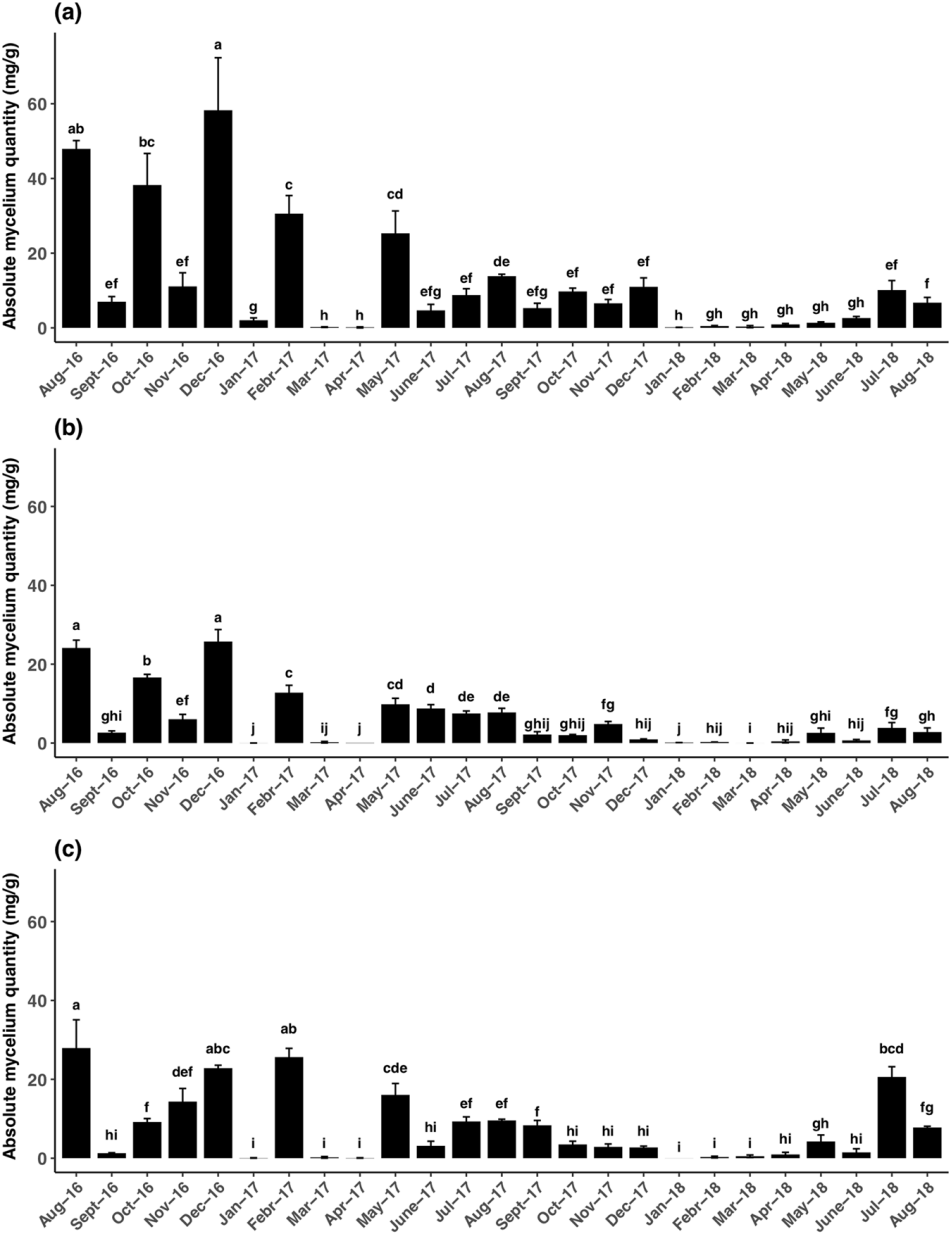
Monthly quantification of *T*. *aestivum* soil DNA from August 2016 to August 2018 for trees II-9 (**a**), III-11 (**b**), and VIII-5 (**c**). Soil DNA is expressed as milligrams of fresh mycelium per gram of dry soil. Error bars represent standard deviation between three replicates. Different letters (**a**–**j**) indicate significant differences between sampling months for each tree (Tukey’s HSD test, p < 0.05).

The *T*. *aestivum* qPCR protocol was applied on soil samples harvested each month under trees II-9, III-11, and VII-5. The *T*. *aestivum* DNA quantity varied each month with a similar trend for all three trees (Fig. 4). It was significantly lower in March/April of 2017 and 2018, although it was higher in August and December 2016 (Fig. 4). Values were higher from August 2016 to August 2017 than from August 2017 to August 2018 (Fig. 4).

**Figure 5:**
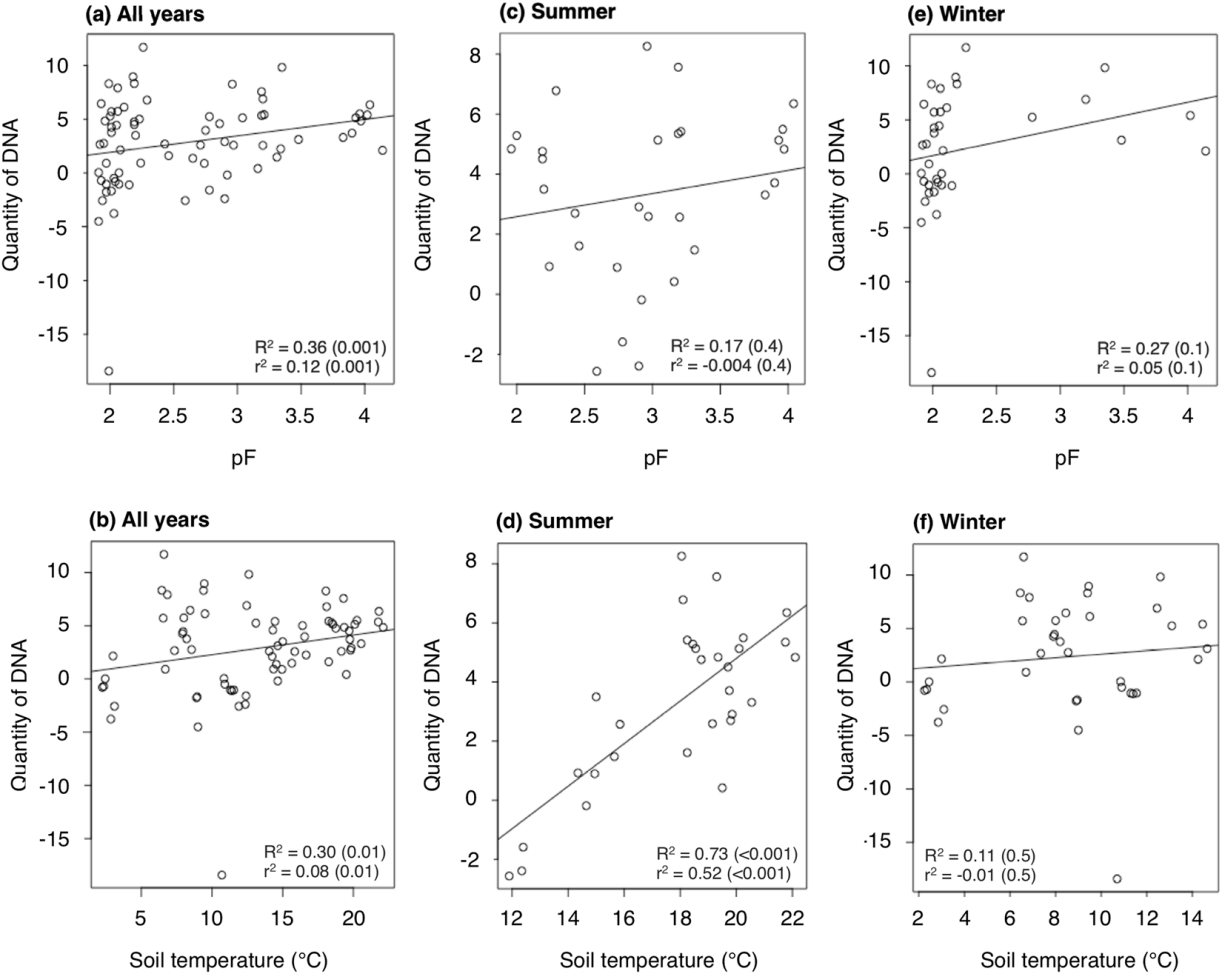
Pearson’s correlation and simple linear regression between soil DNA quantities, pF, and temperature. Temperature and pF data were analysed together for all years (**a**,**b**) and separately for summer (**c**,**d**) and winter (**e**,**f**). Summer corresponds to the period from April to August. Winter corresponds to the period from October to March. The soil DNA quantities were log and square root transformed. R^2^: Pearson’s correlation coefficient; r^2^: simple linear regression’s correlation coefficient.

Pearson’s correlation and simple linear regression analyses showed a significant (p values < 0.05 for both) correlation between soil DNA quantities and pF explaining 36% and 12% of the variability, respectively, when all seasons were analysed together (Fig. 5a). A significant correlation was also found between soil DNA quantities and soil temperatures. These correlations explained 30% and 8% of the variability according to the two analyses (Fig. 5b). No significant correlation was found between pF and soil DNA quantities when the analysis was done separately for summer and winter (Fig. 5c,e; Supplementary Table S6). Temperature data showed a high positive correlation in summer (p value < 0.001) although no correlation was observed in winter (Fig. 5d,f; Supplementary Table S6). The combined effect of pF and temperature tested by factorial ANOVA was not significant (p value = 0.59).

## Discussion

### The ascocarp production reached in this orchard is especially high

The *T*. *aestivum* plantation was not set up to study management techniques; thus, we cannot conclude if the management technique applied here was the best. However, the orchard produced 49 kg per 1500 m^2^ 12 years after planting (about 320 kg/ha equivalent). This production was especially high versus other *T*. *aestivum* truffle grounds. In a plantation of seedlings inoculated with *T*. *melanosporum*, *T*. *aestivum* totally substituted *T*. *melanosporum* 16 years after planting^17^. The maximum of production was about 30 kg/ha. The *T*. *aestivum* production was higher in a 41.5 ha forest in Hungary producing about 104 kg/ha^24^. For *T*. *melanosporum*, one plantation in the nineteen-century and another of the beginning of the 21^st^ century had a maximum production of about 70 kg/ha^2^^ 25^. The production level seen here is thus exceptionally high.

### Temporary wilting point was only reached a few days

The temporary wilting point (pF = 4) seemed to be an important threshold for *T*. *melanosporum* irrigation^26^. Indeed, maintaining the pF below this threshold increased the production compared to non-irrigated controls^26^.

To date the pF was never recorded in a *T*. *aestivum* orchard. Three *C*. *betulus* (trees II-9, III-11 and VIII-5) were selected for soil hydric potential and temperature monitoring. This species was selected among the five tree species because *C*. *betulus* was the most productive according to mean truffle production per plants (Fig. 2). According to these results, the real potential of *C*. *betulus* as host for truffle would deserve further study. The pF was significantly lower for tree VIII-5 than for trees II-8 and III-11 (Fig. 3b). Tree VII-5 was present in the 417 trees/ha density area although the other two trees were localized in the higher density area (833 trees/ha) (Fig. 1). The difference in hydric potential could be explained by the rain interception in 833 trees/ha density as well as by higher water consumption of trees. The rain intercept was well illustrated by the north probe under tree II-9 for which the rains of July 24^th^ 2017 did not allow the pF to reach field capacity although this was seen in the southern probe (Supplementary Fig. S2). High-density plantations for *T*. *aestivum* are often suggested (about 1000 trees/ha), but this needs to be questioned without a drastic tree-pruning strategy. Beside the water availability, density could also impact the production as it was observed in an experimental truffle orchard in Grand-Est region (France) where four densities were implanted (2000, 1000, 666, and 333 trees/ha)^27^. The production started 11 years after plantation in the 2000 trees/ha plantation, but it decreased dramatically a few years ago. In the long term, the lowest density (333 tree/ha) was the most favourable because even though the production started later it continuously increased from 14 to 20 years after planting.

### Monthly variation of soil *T. aestivum* DNA are partly explained by soil temperature and hydric potential

The soil mycelium of ectomycorrhizal species plays a key role in soil nutrient uptake and transfer of carbon from plant to fruiting bodies^28^. Molecular methods have been used to analyse soil DNA of specific ectomycorrhizal fungi to evaluate the ecological and functional impact of a given species on its natural environment^29^. For example, a targeted qPCR protocol has shown that warmer and drier soil conditions reduced the *Lactarius vinosus* soil DNA^30^. A qPCR protocol was designed for quantifying *T*. *melanosporum* and *T*. *magnatum* DNA in the soil^31,32^. In the latter species, it has been shown by a model approach that the soil mycelium was favoured at 20 °C with pF of about 2^33^. The genome of *T*. *aestivum* was recently sequenced^18^, this allowed us to design a specific qPCR protocol. Indeed, the existing protocol using ITS region^21,34^ also amplified *T*. *mesentericum*.

Using this qPCR protocol, we detected a monthly variation of the *T*. *aestivum* soil DNA. This variation correlated with soil temperature and hydric potential. Soil temperature is an important factor regulating microbial activity and shaping the soil microbial community^35^. The effect of temperature on truffle growth has been analysed *in vitro*^36,37^, but our report showed the impact of temperature on truffles in the soil for the first time by a monthly sampling. In our study, the quantity of DNA was low below 5 °C with the highest quantity detected at 6 °C (Supplementary Table S6). *In vitro*, *T*. *melanosporum* responds to cold temperature with specific gene profiling^38^. Our results suggest that *T*. *aestivum* also has a similar machinery to tolerate cold temperatures. Fungal growth rates are typically optimum around 25–30 °C, but the optimum growth temperature varies according to species and isolates^35,39^. In our study, the soil temperature was never above 22 °C. The second highest value of DNA was observed at 20 °C, indicating that *T*. *aestivum* mycelium in the soil can grow between 6 to 20 °C with more growth at higher temperatures. This range is in accordance with data available for other truffle species. Indeed, Iotti *et al*.^33^ determined 20 °C as the optimal temperature for *T*. *magnatum* mycelium growth in the soil.

According to Coleman *et al*.^40^, three patterns of response to water stress could be observed for ectomycorrhizal mycelial growth: growth only occurred in control treatments without hydric stress (Type I). Growth decreases with increasing stress and the maximum growth is always without stress (Type II). The maximum growth does not occur in the control but rather when the mycelium is under hydric stress (Type III). Here, the two highest values of DNA were observed in non-stressed conditions (pF = 2.2) and in a very high stress condition (pF = 5.9). There was also a linear correlation between pF and DNA quantity (Fig. 5). Thus, we conclude that *T*. *aestivum* is a type III species such as *Terfezia boudieri* and *Picoa lefebvrei* two desert truffles^41^. *T*. *aestivum* is drought tolerant because it can grow above pF 4.3^40^. On the contrary, *T*. *magnatum* seems to belong to type I since the optimal water potential is considered as being at pF around 2^33^. Both truffle species have different ecological requirements. *T*. *magnatum* is most of the time harvested in riparian conditions while *T*. *aestivum* is harvested in less humid soil conditions. These results have practicable implications for truffle growers: irrigation must be triggered at very different soil water potentials for both species.

Temperature and pF only partially explain the content of soil DNA variability (36% to 30% according the analyses; Fig. 5). Other factors therefore could influence summer truffle soil DNA, but they were not analysed in this study. *T*. *aestivum* could enter into competition with other naturally occurring ectomycorrhizal fungi.

According to Stobbe *et al*.^11,12^, *T*. *aestivum* can fruit all the year, and the only two months without truffles are March and April. Interestingly, we found that months with less soil DNA were also March and April for 2017 and 2018. Unfortunately, because of her personal schedule, the owner of the orchard only harvested ascocarps from May to August, which did not allow us to detect a putative link between ascocarp production and soil DNA.

The quantity of soil *T*. *aestivum* DNA decreased from 2016/2017 to 2018. In parallel, the production of ascocarps in 2018 is lower than in 2017 with a decrease of about 88%. For *T*. *magnatum* and *T*. *melanosporum*, the quantity of DNA is higher in productive versus non-productive plots^29,31,42^. Thus, it is tempting to link the decrease in ascocarp production to the decrease in soil DNA. This orchard will continue to be analysed to gain more information about the putative link between ascocarp production and quantity of soil DNA.

## Material and Methods

### Experimental site

The *T*. *aestivum* orchard is located at Meux (45.442921, −0.358027) in the Nouvelle-Aquitaine region of France covering 1500 square meters. All the area interested by the plantation is characterized by a calcareous chalky. Wheat occupied the field before the plantation. After wheat harvest, the soil was returned and then tilled with rotary harrow. In 2004, 34 *Corylus avellana*, 10 *Carpinus betulus*, 10 *Tilia cordata*, 10 *Quercus pubescens*, and 10 *Pinus nigra* seedlings inoculated with *T*. *aestivum* were planted 4 m apart in the line and 6 m between lines (Fig. 1). Seedlings of the different species were planted randomly. In 2005, 43 additional *C*. *avellana* seedlings inoculated with *T*. *aestivum* were implanted between lines 7 to 17 (Fig. 1). Thus, there are two densities in the orchards: a high density (4 × 3 m equivalent to 833 trees per hectare) and a lower density (4 × 6 m equivalent to 417 trees per hectare). From 2004 to 2006, weeds were controlled by a monthly soil tilling with a rotovator and the proximity of trees was cleaned with a pickaxe. There was no soil tilling from 2007 to 2011; weeds were controlled using a brush-cutter and a few chemical treatments with glufosinate-ammonium (Basta, Bayer). Spores have been added every year since 2008 (from 5 to 100 g of crushed ascocarps). The production started in 2009, and the ascocarps were harvested with a trained dog from May to August. All harvested truffles were assigned to the closest tree. Slight pruning was realized since 2009 to avoid total closure of the orchard. The soil was tilled with a rotovator in September 2012, 2015, and 2017 to avoid soil compaction. In summer, the orchard was irrigated with potable water according to empirical criteria.

### Development of a *T. aestivum* qPCR specific protocol

The *T*. *aestivum* genome has recently been sequenced^18^. In the U.S. Department of Energy Joint Genome Institute (DOE-JGI) genome browser (https://genome.jgi.doe.gov/Tubae1), *T*. *aestivum* orphan genes (i.e. genes present only in the *T*. *aestivum* genome and absent in the other genomes) were identified using the cluster analysis considering eight Pezizomycete species: *Ascobolus immersus*, *Choiromyces venosus*, *Morchella importuna*, *Pyronema confluens*, *Terfezia boudieri*, *T*. *aestivum*, *T*. *magnatum*, and *T*. *melanosporum*. Only clusters with one single gene copy in *T*. *aestivum* were considered. Two gene models (clusters 17,691 and 17,699) with numbers GSTUAT00001921001 (proteinId 4119) and GSTUAT00007989001 (proteinId 4108), respectively, were randomly selected. The primers TuGM4119F/TuGM4119R and TuGM4108F/TuGM4108R were then designed with Amplify4 v1.0 software in these gene models (Table 1). Primers were designed for use with qPCR via the SYBR® Green dye.

**Table 1:** Primers used in this study.

Primers	Sequence (5′-3′)	References	Thermal program used in PCR
TuGM4108F	GCG GTA CCC GGG AAT ATG GT	This study	94 °C, 4 min, 30 cycles (94 °C, 30 s; 65 °C, 30 s; 72 °C, 45 s), 72 °C, 5 min
TuGM4108R	CAG CGA AGC TCA GGT GTG GA		
TuGM4119F	AAA AAC AGG CCC ACA TCA AG	This study	94 °C, 4 min, 30 cycles (94 °C, 30 s; 60 °C, 30 s; 72 °C, 45 s), 72 °C, 5 min
TuGM4119R	GGC TCA CGG TGA TTG ATT CT		
ITS1F	CTT GGT CAT TTA GAG GAA GTA A	White *et al*.^43^; Gardes & Bruns^44^	94 °C, 4 min, 35 cycles (94 °C, 30 s; 55 °C, 30 s; 72 °C, 45 s), 72 °C, 5 min
ITS2	GCT GCG TTC TTC ATC GAT GC		
TuITS1	ACC ACA GCT GCG TAC AAT GCC	Luis^45^	94 °C, 4 min, 30 cycles (94 °C, 30 s; 65 °C, 30 s; 72 °C, 45 s), 72 °C, 5 min
TuITS4	GAT CCG AGG TCA AAC CTG ACG		

The specificity of the two gene model primers was tested via PCR using 13 DNA samples from a first set of *Tuber* species ascocarps (Supplementary Table S2a): *T*. *aestivum*, *T*. *mesentericum*, *T*. *magnatum*, *T*. *borchii*, *T*. *melanosporum*, *T*. *brumale*, and *T*. *indicum*. The DNA of each sample was extracted using the DNeasy™ Plant Mini Kit (Qiagen SA, Courtaboeuf, France). A second set of DNA was extracted from 16 DNA samples of dried *T*. *aestivum* ascocarps of different geographical locations (Supplementary Table S2b). The universal fungal primers ITS1F and ITS2^43,44^ (Table 1) were used to check that the DNA was suitable for PCR amplification (Supplementary Figs S7a and S8a). The PCR amplification was performed in a 10-µL mixture composed of 2X REDExtract-N-Amp™ PCR ReadyMix™ (5 µL), 10 µM primer forward (0.4 µL), 10 µM primer reverse (0.4 µL), 16 mg/mL bovine serum albumin (0.35 µL), water (1.85 µL), and 1/10 diluted DNA (2 µL). PCR reactions were carried out on a C1000 Touch™ Thermal Cycler (Bio-Rad, Schiltigheim, France) with denaturation at 94 °C for 4 min followed by 35 cycles of denaturation at 94 °C for 30 s, annealing at 55 °C for 30 s, extension at 72 °C for 45 s, and a final extension at 72 °C for 5 min.

To confirm that the ascocarps belonged to *T*. *aestivum*, PCR amplifications were performed with the *T*. *aestivum* specific primers TuITS1 and TuITS4^45^ (Table 1; Supplementary Figs S7b and S8b). The PCR mixture was the same as those used for the ITS universal primers with the following thermal profile: denaturation at 94 °C for 4 min, 30 cycles of denaturation at 94 °C for 30 s, annealing at 65 °C for 30 s, extension at 72 °C for 45 s, and a final extension at 72 °C for 5 min.

The first set of 13 DNA samples were amplified with primers TuGM4119f/TuGM4119r and TuGM4108f/TuGM4108r (Table 1) by PCR with the same PCR mixture described above and according to the following conditions: denaturation at 94 °C for 4 min, 30 cycles of denaturation at 94 °C for 30 s, annealing at 65 °C for 30 s, extension at 72 °C for 45 s, and a final extension at 72 °C for 5 min. All PCR products were run on 2% agarose gel and visualized with a UV trans illuminator (*Geldoc™ Universal Hood II*, *Biorad)* after ethidium bromide staining.

According to the direct PCR results, real-time PCR amplification with SYBR® Green dye was then carried out on both sets of DNA with primers TuGM4108f/TuGM4108r only. The 20 µL of qPCR mixture was composed of Takyon™ No Rox SYBR® Core Kit (16.4 µL), 10 µM primer forward (0.8 µL), 10 µM primer reverse (0.8 µL), and template DNA (2 µL). The qPCR reactions were performed with a StepOnePlus™ Real-Time PCR System machine provided with the StepOne software v. 2.3 (Life Technologies, Applied Biosystem) on 96-well plates with carry over prevention at 50 °C for 2 min, Takyon™ activation at 95 °C for 3 min, 40 cycles of denaturation at 95 °C for 5 s, annealing at 60 °C for 15 s, and extension at 72 °C for 15 s.

### Soil sampling, treatment, and DNA extraction

Three highly producing *C*. *betulus* were selected and equipped with two tensiometers (TEROS-21, Meter Group). These trees were randomly selected from the best producing trees (Supplementary Table S1). Two trees (II-9 and III-11) were selected from the 833 trees/ha area and tree VIII-5 was sampled from the 417 trees/ha area (Fig. 1). Tensiometers were installed 1 m from tree trunks: one at the north and another at the south—both at 12 cm depth. The TEROS-21 records soil water potential and soil temperature. Soil water potential is expressed as pF corresponding to the logarithm of the suction force expressed as centimetre height in a water column. The probes were installed on August 30, 2016. Soil sampling was performed every month 1 m from the trunk at the four cardinal points (North, West, East and South) under all the three trees at a depth of 5–10 cm. After sampling, the soil samples were immediately sent to the laboratory. They were initially dried separately about 2 hours at 40 °C and sieved using a 2-mm mesh and then stored at −20 °C until DNA extraction. The DNA of each sample was extracted using the DNeasy PowerSoil™ DNA Isolation Kit (Qiagen SA, Courtaboeuf, France) from 0.25 g of sieved soil. The extracted DNA was stored at −20 °C until analysis. For the molecular analysis described below, the four DNA samples around one tree were pooled to obtain one sample per tree and per sampling time.

### Quantification of soil DNA

A standard curve was created as described in Parladé *et al*.^32^ by mixing a known amount of soil harvested in a field close to the truffle orchard with a known amount of fresh *T*. *aestivum* ascocarp. The absence of *T*. *aestivum* in the soil was first controlled by qPCR and the ascocarp was then ground in a mortar with liquid nitrogen. The DNA was extracted from the mixture in the same way as other soils; serial 10-fold dilutions were done to obtain a standard curve.

The soil *T*. *aestivum* DNA was measured with real-time PCR with SYBR® Green dye. The TuGM4108 primers (Table 1) were used to amplify the *T*. *aestivum* model gene in soil samples as described above. qPCR was performed on 96-well plates with three replicates for each sample, standard, and negative control. One sample corresponded to the mix of the four soil DNA extractions around one tree at one sampling date. The qPCR mixture and the thermal conditions were the same as those described above for the specificity assay. The software calculated the absolute quantity of *T*. *aestivum* in soil samples by interpolating the threshold cycle (Ct) values on the standard curve (R^2^ = 0.99; Eff = 96.02%).

### Statistical analysis

The difference in daily pF and temperature between the north and south probes within the three productive trees and difference of daily pF and temperature (average between north and south probes) between the three productive trees were analysed by pairwise comparison using a Wilcoxon rank sum test (p < 0.05). The differences between qPCR replicates were analysed with multifactor analysis of variance (ANOVA), differences of *T*. *aestivum* DNA quantifications between sampling months were detected with Tukey’s HSD test (p < 0.05).

In order to compare the mean production per tree species, a subsampling was realized for *C*. *avellana* (Supplementary Fig. S1). Indeed, 34*C*. *avellana* were implanted in 2004 and 43 in 2005 although for all other species 10 plants were implanted in 2004. A random subsampling of 10*C*. *avellana* was realized 10 times among the 34 plants implanted in 2004 using *sample* function in R. *Boxplot* function in R was used to visualized the mean value obtained for the 10 subsampling. The differences were statistically tested with Tukey’s HSD test.

The relationships between *T*. *aestivum* soil DNA quantities and soil parameters (pF and temperature) were tested using two methods: (1) linear correlation using Pearson’s method with correlation coefficient and p-value calculation using *cor*.*test* function in *stats* R package; and (2) simple linear regression with *lm* function of the *stats* R package. The combined effect of pF and temperature was tested by a factorial ANOVA using *aov* function in R. Soil DNA quantities were log and square root transformed to fit normality and improve variance equality.

## Supplementary information

Supplementary Figures

Supplementary Tables

## Data Availability

All the data are included in the main manuscript and supplementary informations. The *Tuber aestivum* genome is available at the Joint Genome Institute website: https://genome.jgi.doe.gov/Tubae1.

## References

[1] Smith, S. E. & Read, D. J. *Mycorrhizal symbiosis*. 3rd edn, Academic Press (2008).

[2] Clemmensen KE (2013). Roots and associated fungi drive long‐term carbon sequestration in boreal forest. Science.

[3] Murat C (2015). Forty years of inoculating seedlings with truffle fungi: past and future perspectives. Mycorrhiza.

[4] Bonito G, Gryganskyi AP, Trappe JM, Vilgalys R (2010). A global meta-analysis of Tuber ITS rDNA sequences: species diversity, host associations and long-distance dispersal. Mol. Ecol..

[5] Bonito G (2013). Historical biogeography and diversification of truffles in the Tuberaceae and their newly identified Southern hemisphere sister lineage. PLoS ONE.

[6] Ceruti A, Fontana A, Nosenzo C (2003). Le specie europee del genere Tuber, una revisione storica. Regione Piemonte, Museo regionale di Scienze Naturali de Torino, Torino.

[7] Boa, E. *Wild edible fungi: a global overview of their use and importance to people*, Vol 17, Non Wood Forest Products, FAO, Rome, 147 (2004).

[8] Mello A, Murat C, Bonfante P (2006). Truffles: much more than a prized and local fungal delicacy. FEMS Microbiol. Lett..

[9] Zambonelli, A., Iotti, M. & Murat, C. True Truffle (Tuber spp.) in the World. Soil ecology, systematics and biochemistry. *Soil Biology*. (Vol. 47. Springer, Cham, Switzerland, 2016).

[10] Reyna S, Garcia-Barreda S (2014). Black truffle cultivation: a global reality. For. Syst..

[11] Stobbe U (2012). Spatial distribution and ecological variation of re-discovered German truffle habitats. Fungal Ecol..

[12] Stobbe U (2013). Potential and limitations of Burgundy truffle cultivation. App. Microbiol. & Biotechnol..

[13] Bagi, I., Fekete, O. Identification of *Tuber aestivum* habitats in the South Caucasus, Azerbaidjan. In *Communication at the 2nd congress of the Tuber aestivum/uncinatum*, European Scientific Group, Juva, 20–22 Aug 2010 (2010).

[14] Chevalier, G. & Frochot, H. *La truffe de Bourgogne*. Pétrarque, Levallois-Perret (2002).

[15] Molinier, V., Peter, M., Stobbe, U. & Egli, S. The Burgundy truffle (*Tuber aestivum* syn. *uncinatum*):a truffle species with wide habitat range over Europe. In Zambonelli, A., Iotti, M. & Murat, C. (eds) *True Truffle (Tuber spp*.*) of the World*. *Soil biology*. Vol 47. (Springer, Cham, Switzerland, 87–103, 2016).

[16] Robin, C., Goutal-Pousse, N. & Le Tacon, F. Soil characteristics for *Tuber aestivum* (Syn. *T. uncinatum*). In Zambonelli, A., Iotti, M. & Murat, C. (eds) *True Truffle (Tuber spp*.*) of the World*. *Soil biology*. (Vol 47. Springer, Cham, Switzerland, 87–103, 2016).

[17] Molinier V (2013). Monitoring the fate of a 30-year-old truffle orchard in Burgundy: from Tuber melanosporum to Tuber aestivum. Agrofor. Syst..

[18] Murat C (2018). Pezizomycetes genomes reveal the molecular basis of ectomycorrhizal truffle lifestyle. Nat. Ecol. Evol..

[19] Molinier Virginie, Murat Claude, Morin Emmanuelle, Gollotte Armelle, Wipf Daniel, Martin Francis (2013). First Identification of Polymorphic Microsatellite Markers in the Burgundy Truffle, Tuber aestivum (Tuberaceae). Applications in Plant Sciences.

[20] Molinier V (2016). Fine-scale genetic structure of natural Tuber aestivum sites in southern Germany. Mycorrhiza.

[21] Gryndler M (2013). Tuber aestivum Vittad. mycelium quantified: advantages and limitations of a qPCR approach. Mycorrhiza.

[22] Gryndler M (2017). Truffle biogeography - a case study revealing ecological niche separation of different Tuber species. Ecol. Evol..

[23] Benucci, G. M. *et al*. Taxonomy, biology and ecology of *Tuber macrosporum* Vittad. and *Tuber mesentericum* Vittad. In Zambonelli, A., Iotti, M. & Murat, C. (eds) *True Truffle (tuber spp*.*) of the World*. (Vol 47, Soil biology, Springer, Cham, Switzerland, 87–103, 2016).

[24] Büntgen U (2017). New Insights into the Complex Relationship between Weight and Maturity of Burgundy Truffles (*Tuber aestivum*). PLoS ONE.

[25] Le Tacon F (2016). Certainties and uncertainties about the life cycle of the Périgord black truffle (*Tuber melanosporum* Vittad.). Ann. For. Sci..

[26] Le Tacon F, Delmas J, Gleyze R, Bouchard D (1982). Influence du régime hydrique du sol et de la fertilisation sur la fructification de la truffe noire du Périgord (*Tuber melanosporum* Vitt.) dans le sud-est de la France, Acta Oecologica. Oecologia appl..

[27] Pousse, J. S., Robin, C., Werhlen, L. & Frochot, H. Plantation density of hazel trees (*Corylus avellana*) influences the precocity and kinetics of Burgundy truffle (*Tuber aestivum*) production. *Österr*. *Z*. *Pilzk*. **19** (2008).

[28] Le Tacon F (2013). Carbon transfer from the host to Tuber melanosporum mycorrhizas and ascocarps followed using a 13C pulse-labeling technique. PLoS ONE.

[29] Parladé, J., De la Varga, H. & Pera, J. Tools to trace truffles in soil. In Zambonelli, A., Iotti, M. & Murat, C. (eds) *True Truffle (Tuber spp*.*) in the World*. *Soil Biology*. (Vol. 47. Springer, Cham, Switzerland, 249–266, 2016).

[30] Castaño C (2016). Soil drying procedure affects the DNA quantification of Lactarius vinosus but does not change the fungal community composition. Mycorrhiza.

[31] Iotti M (2012). Development and validation of a real-time PCR assay for detection and quantification of Tuber magnatum in soil. BMC Microbiol..

[32] Parladé J, De la Varga H, De Miguel AM, Sáez R, Pera J (2013). Quantification of extraradical mycelium of Tuber melanosporum in soils from truffle orchards in northern Spain. Mycorrhiza.

[33] Iotti M, Leonardi P, Vitali G, Zambonelli A (2018). Effect of summer soil moisture and temperature on the vertical distribution of Tuber magnatum mycelium in soil. Biol Fertil Soils.

[34] Gryndler M (2011). Detection of summer truffle (*Tuber aestivum Vittad*.) in ectomycorrhizae and in soil using specific primers. FEMS Microbiol. Lett..

[35] Pietikäinen J, Pettersson M, Bååth E (2005). Comparison of temperature effects on soil respiration and bacterial and fungal growth rates. FEMS Microbiol. Ecol..

[36] Sanchez F, Honrubia M, Torres P (2001). Effects of pH, water stres and temperature on *in vitro* cultures of ectomycorrhizal fungi from Mediterranean forests. Cryptogam. Mycol..

[37] Nadim M (2016). Effects of some environmental parameters on Mycelia Growth of Finnish truffle Tuber Maculatum. Int. J. Eng. & Appl. Sci..

[38] Zampieri E (2011). The Perigord black truffle responds to cold temperature with an extensive reprogramming of its transcriptional activity. Fungal Genet. Biol..

[39] Cline ML, France RC, Reid CP (1987). Intraspecific and interspecific growth variation of ectomycorrhizal fungi at different temperatures. Can. J. Bot..

[40] Coleman MD, Bledsoe CS, Lopushinsky W (1989). Pure culture response of ectomycorrhizal fungi to imposed water stress. Can. J. Bot..

[41] Navarro-Ródenas A, Lozano-Carrillo MC, Pérez-Gilabert M, Morte A (2011). Effect of water stress on *in vitro* mycelium cultures of two mycorrhizal desert truffles. Mycorrhiza.

[42] Suz LM, Martin MP, Oliach D, Fischer RC, Colinas C (2008). Mycelial abundance and other factors related to truffle productivity in Tuber melanosporum–Quercus ilex orchards. FEMS Microbiol. Lett..

[43] White, T. J. *et al*. *Amplification and* direct sequencing of fungal ribosomal *RNA genes for phylogenetics*. *PCR Protocols*. *A Guide to Methods and Applications* (Innis, M. A., Gelfand, D. H. & Sninsky, J. J. eds) (Academic Press, San Diego, 315–322, 1990).

[44] Gardes M, Bruns TD (1993). ITS primers with enhanced specificity for Basidiomycetes: application to identification of mycorrhizae and rusts. Mol. Ecol..

[45] Luis, P. Caractérisation de la diversité génétique des populations chez *Tuber uncinatum* et *Tuber melanosporum*. DEA de l’université Claude Bernard, Lyon I-France, **47** (2000).

