# Supplementary material for: Soil temperature and hydric potential influences the monthly variations of soil Tuber aestivum DNA in a highly productive orchard
Source: Sci Rep. 2019 Sep 10;9:12964. doi: 10.1038/s41598-019-49602-2 (PMC6736833; doi:10.1038/s41598-019-49602-2)
Supplement: Supplementary file 1 — Supplementary Figures [file 41598_2019_49602_MOESM1_ESM.pdf]

Soil temperature and hydric potential influences the monthly variations of soil *Tuber aestivum* DNA in a highly productive orchard

Flora Todesco, Simone Belmondo, Yoann Guignet, Liam Laurent, Sandrine Fizzala, François Le Tacon, Claude Murat

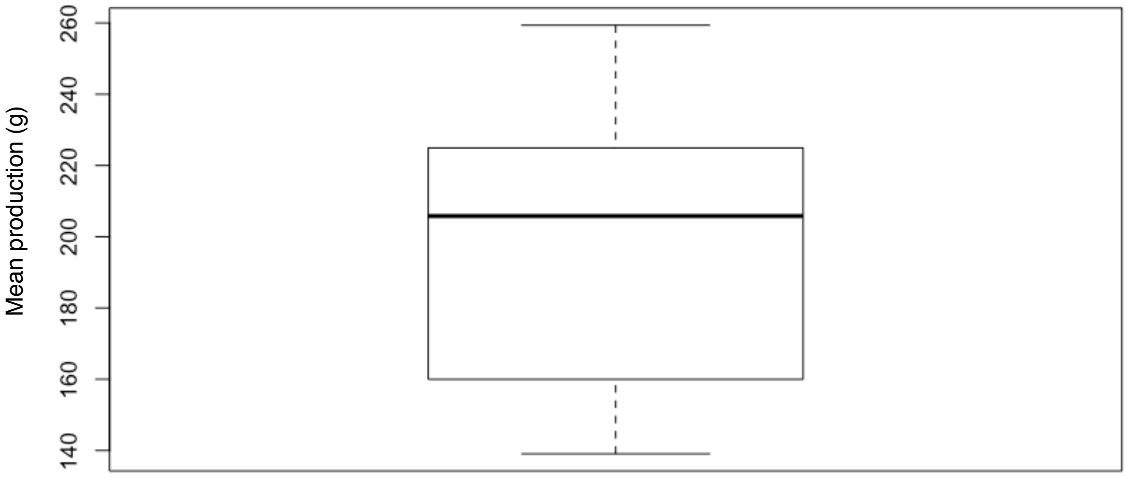

Supplementary Fig. S1 Boxplot obtained with the 10 subsampled *C. avellana*

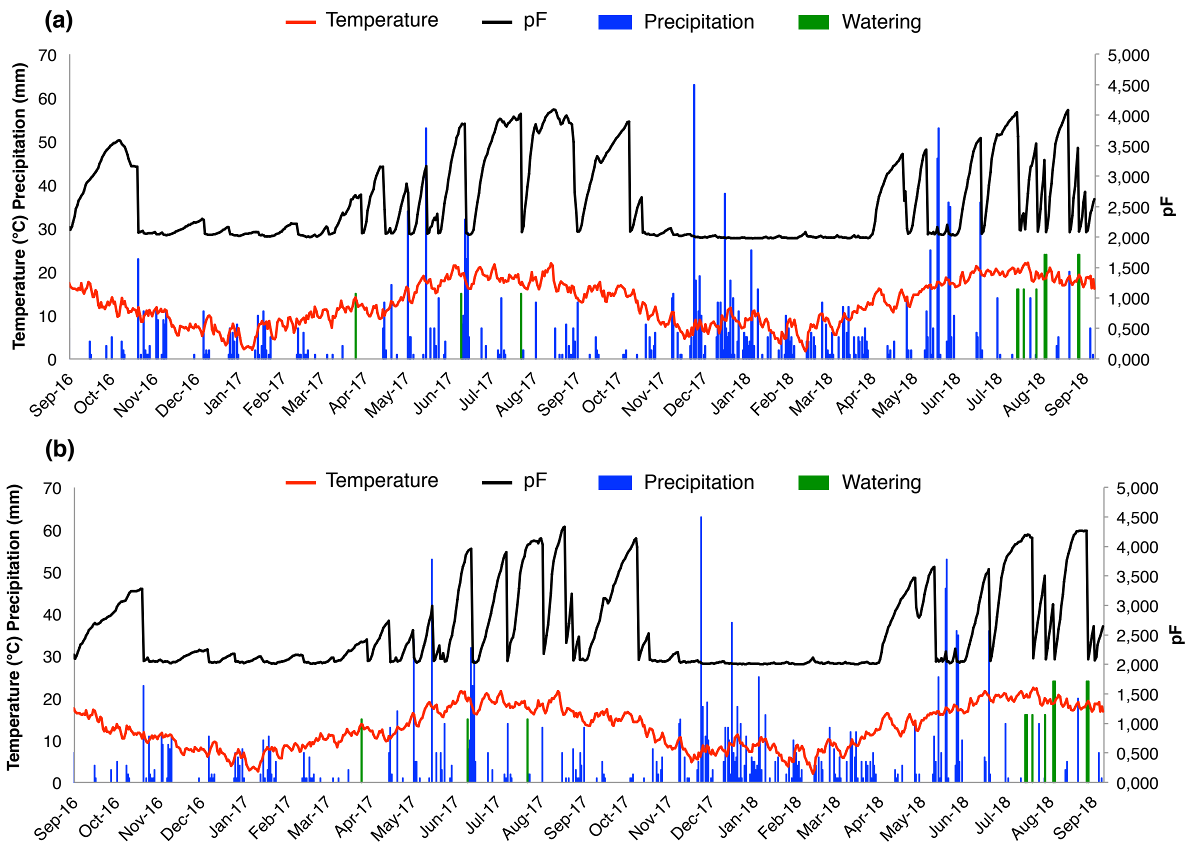

Supplementary Fig. S2 Graphic representation of daily rainfall, pF (i.e. soil hydric potential), and temperature measured from September 2016 to September 2018 at the north (a) and south (b) probes under tree II-9. Red lines, temperatures (°C); black lines, pF; blue bars, daily rainfall (in mm); and green bars, watering.

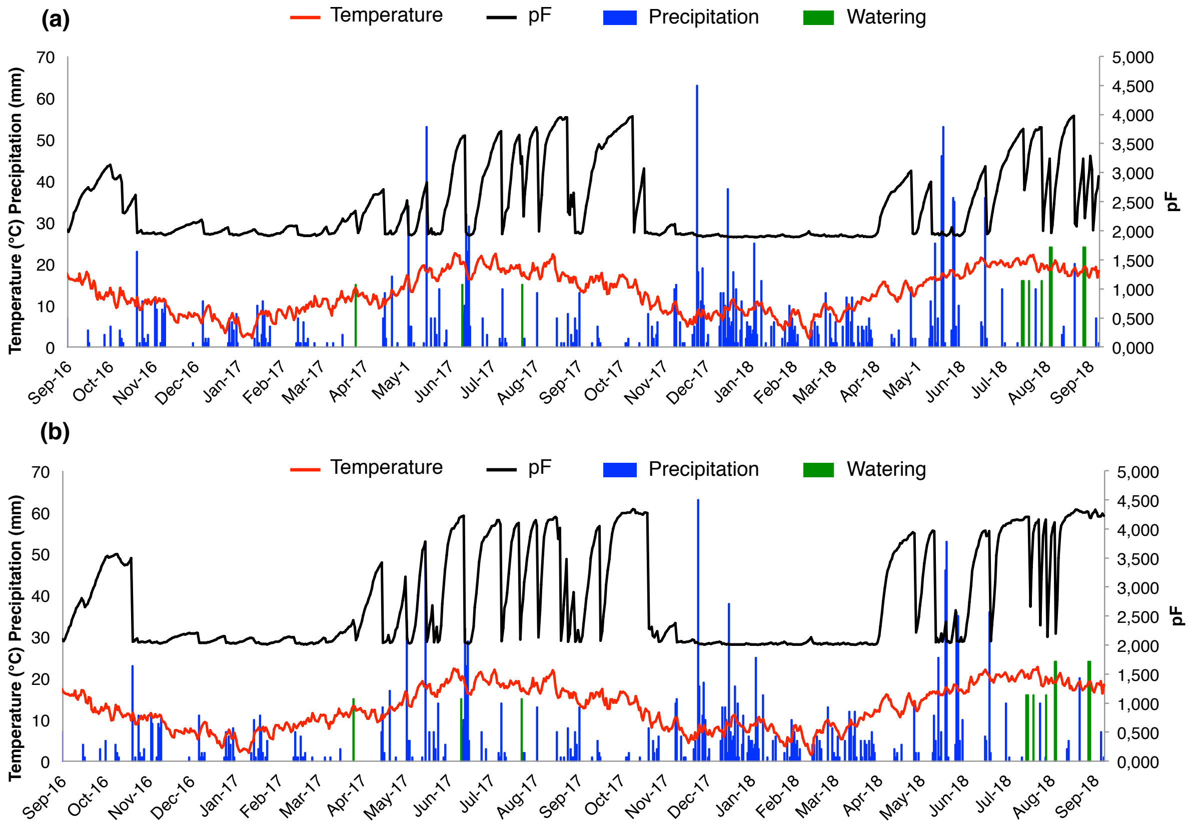

Supplementary Fig. S3 Graphic representation of daily rainfall, pF (i.e. soil hydric potential), and temperature from September 2016 to September 2018 at the north (a) and south (b) probes under tree III-11. Red lines, temperatures (°C); black lines, pF; blue bars, daily rainfall (in mm); and green bars, watering.

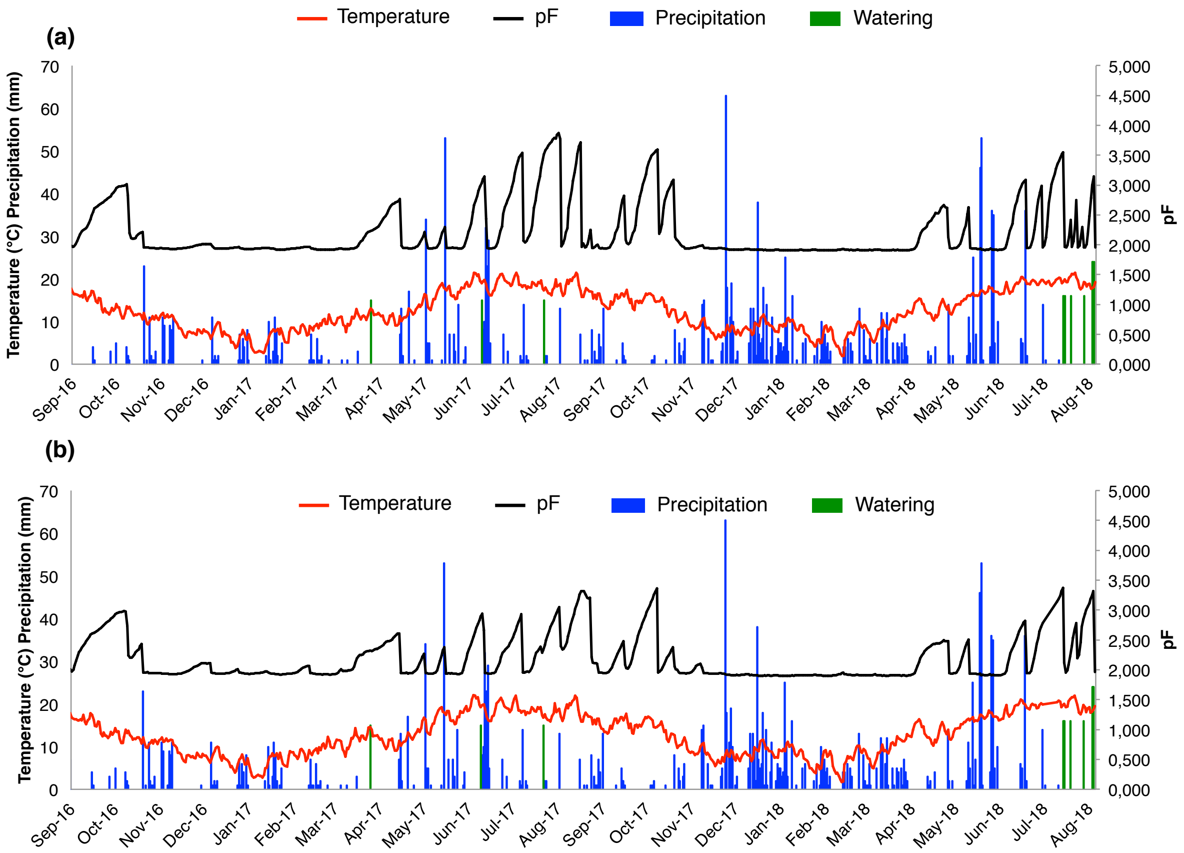

Supplementary Fig. S4 Graphic representation of daily rainfall, pF (i.e. soil hydric potential), and temperature from September 2016 to September 2018 at the north (a) and south (b) probes under tree VIII-5. Red lines, temperatures (°C); black lines, pF; blue bars, daily rainfall (in mm); and green bars, watering.

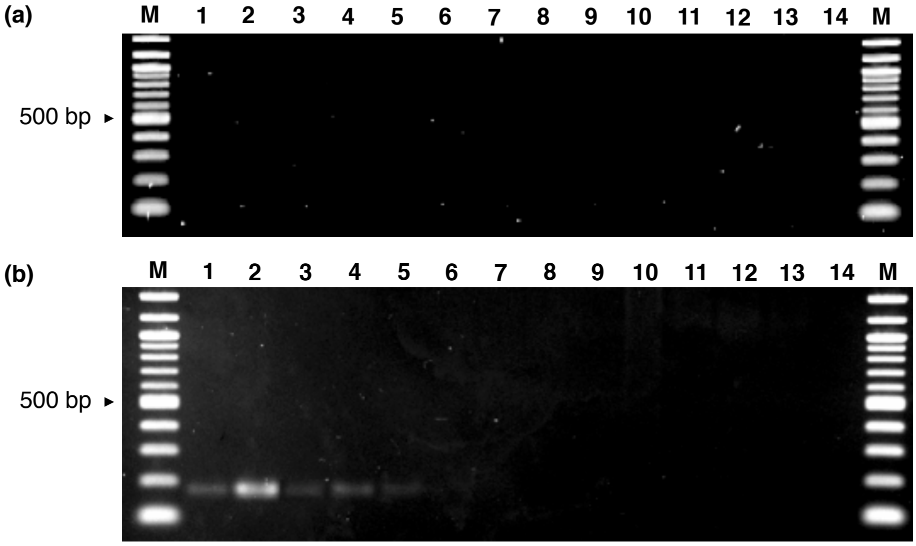

Supplementary Fig. S5 PCR amplifications of DNA from 13 ascocarps of different *Tuber* species with the model gene primers TuGM4119f/TuGM4119r (a) and with the model gene primers TuGM4108f/TuGM4108r (b). Lane M, 100 bp DNA ladder marker (NEB); lanes 1-5, *Tuber aestivum*; lanes 6-8, *Tuber mesentericum*; lane 9, *Tuber magnatum*; lane 10, *Tuber borchii*; lane 11, *Tuber melanosporum*; lane 12, *Tuber brumale*; lane 13, *Tuber indicum*; and lane 14, negative control.

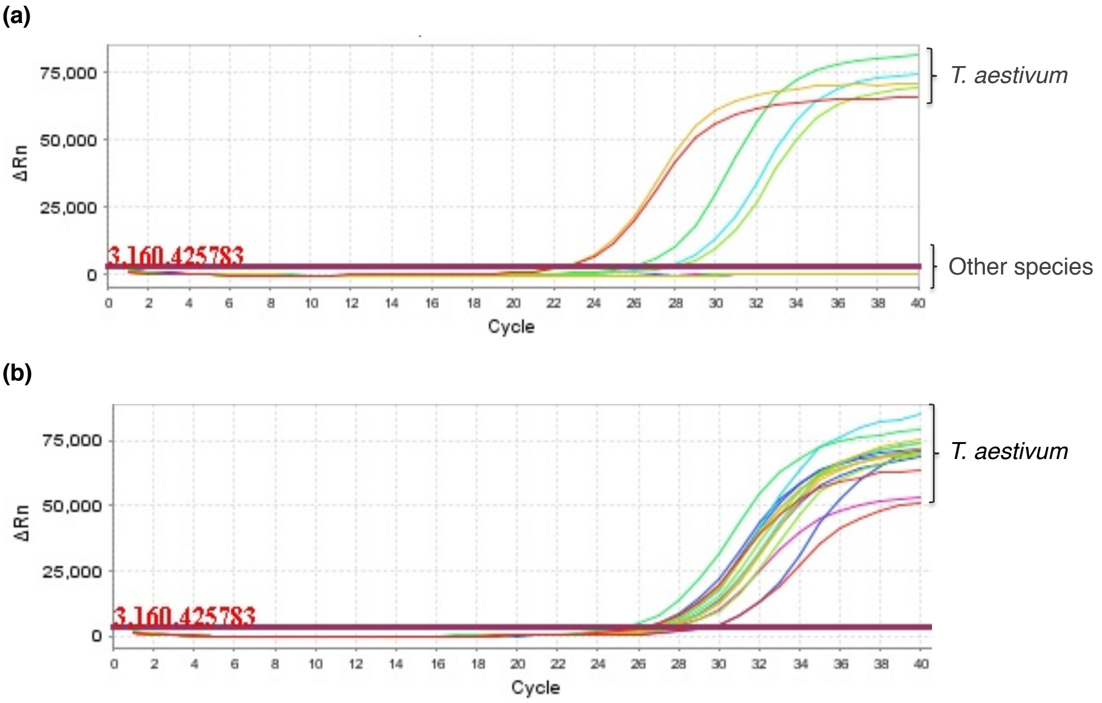

Supplementary Fig. S6 qPCR amplifications of 13 DNA samples from different *Tuber* spp. (a) and 16 DNA from *Tuber aestivum* ascocarps of different geographical locations (b) with the model gene primers TuGM4108f/TuGM4108r. The purple line represents the Threshold Cycle allowing one to conclude sample positivity. Only the 5 *T. aestivum* DNA was amplified among the different *Tuber* spp.; all of the 16 DNA from *Tuber aestivum* ascocarps of different geographical locations amplify.

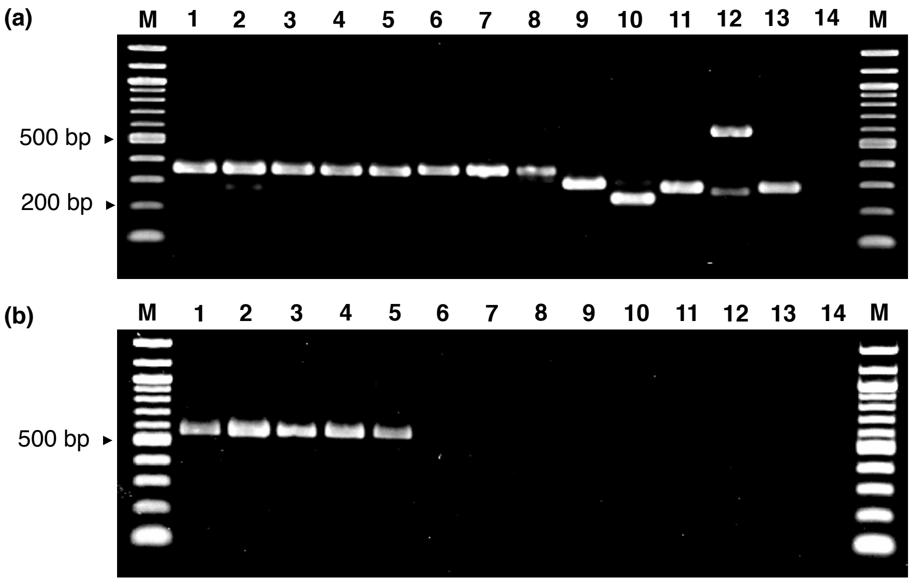

Supplementary Fig. S7 PCR amplifications of DNA from 13 ascocarps of different *Tuber* species with universal fungal primers ITS1F/ITS2 (a) and with the *Tuber aestivum*-specific primers TuITS1/TuITS4 (b). Lane M, 100 bp DNA ladder marker (NEB); lanes 1-5, *Tuber aestivum*; lanes 6-8, *Tuber mesentericum*; lane 9, *Tuber magnatum*; lane 10, *Tuber borchii*; lane 11, *Tuber melanosporum*; lane 12, *Tuber brumale*; lane 13, *Tuber indicum*; and lane 14, negative control.

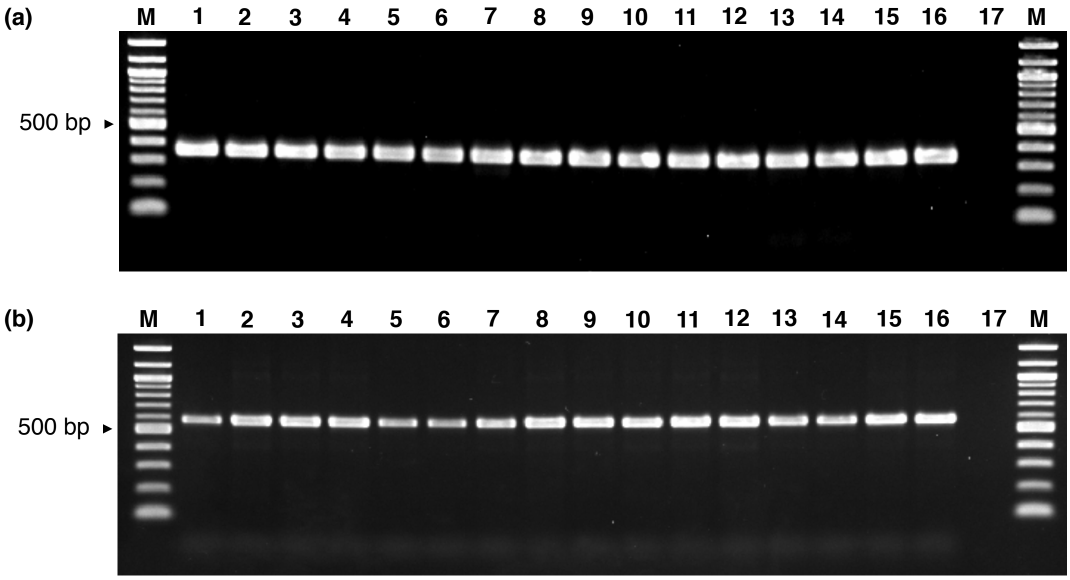

Supplementary Fig. S8 PCR amplifications of DNA from 16 *Tuber aestivum* ascocarps of different geographical locations with the universal fungal primers ITS1F/ITS2 (a) and with the *Tuber aestivum*-specific primers TuITS1/TuITS4 (b). Lane M, 100 bp DNA Ladder marker (NEB); lane 1, Spain (Soria); lanes 2-3, France (Drôme); lanes 4-5, Great Britain; lanes 6-7, Hungary; lane 8, France (Lot); lanes 9-12, France (Hérault); lanes 13-15, France (Vaucluse); lane 16, Romania; and lane 17, negative control.
